# The ACCEPT-study: design of an RCT with an active treatment control condition to study the effectiveness of the Dutch version of PEERS® for adolescents with autism spectrum disorder

**DOI:** 10.1186/s12888-020-02650-9

**Published:** 2020-06-01

**Authors:** B. J. van Pelt, S. Idris, G. Jagersma, J. Duvekot, A. Maras, J. van der Ende, N. E. M. van Haren, K. Greaves-Lord

**Affiliations:** 1 Department of Child and Adolescent Psychiatry/Psychology, Erasmus MC-Sophia, Wytemaweg 8, 3015 CN Rotterdam, The Netherlands; 2 Yulius Organization for Mental Health, Burg, De Raadtsingel 93c, 3311 JG Dordrecht, The Netherlands; 3 Department of Psychiatry, Clinical Psychology Unit, Faculty of Medicine, Universiti Teknologi MARA, 68100 Batu Caves, Shah Alam, Selangor Malaysia; 4 Autism Team Northern-Netherlands, Jonx, department of (Youth) Mental Health and Autism of Lentis Psychiatric Institute Groningen, Groningen, The Netherlands; 5 Department of Clinical Psychology and Experimental Psychopathology, University of Groningen, Groningen, The Netherlands

**Keywords:** Autism, RCT, Peers®, Social skills, Adolescents

## Abstract

**Background:**

Social skills interventions are commonly deployed for adolescents with autism spectrum disorder (ASD). Because effective and appropriate social skills are determined by cultural factors that differ throughout the world, the effectiveness of these interventions relies on a good cultural fit.

Therefore, the ACCEPT study examines the effectiveness of the Dutch Program for the Education and Enrichment of Relational Skills (PEERS®) social skills intervention.

**Methods/design:**

This study is a two-arm parallel group randomized controlled trial (RCT) in which adolescents are randomly assigned (after baseline assessment) to one of two group interventions (PEERS® vs. active control condition). In total, 150 adolescents are to be included, with multi-informant involvement of their parents and teachers. The ACCEPT study uses an active control condition (puberty psychoeducation group training, focussing on social-emotional development) and explores possible moderators and mediators in improving social skills. The primary outcome measure is the Contextual Assessment of Social Skills (CASS). The CASS assesses social skills performance in a face to face social interaction with an unfamiliar, typically developing peer, making this a valuable instrument to assess the social conversational skills targeted in PEERS®. In addition, to obtain a complete picture of social skills, self-, parent- and teacher-reported social skills are assessed using the Social Skills improvement System (SSiS-RS) and Social Responsiveness Scale (SRS-2). Secondary outcome measures (i.e. explorative mediators) include social knowledge, social cognition, social anxiety, social contacts and feelings of parenting competency of caregivers. Moreover, demographic and diagnostic measures are assessed as potential moderators of treatment effectiveness. Assessments of adolescents, parents, and teachers take place at baseline (week 0), intermediate (week 7), post intervention (week 14), and at follow-up (week 28).

**Conclusion:**

This is the first RCT on the effectiveness of the PEERS® parent-assisted curriculum which includes an active control condition. The outcome of social skills is assessed using observational assessments and multi-informant questionnaires. Additionally, factors related to social learning are assessed at several time points, which will enable us to explore potential mediators and moderators of treatment effect.

**Trail registration:**

Dutch trail register NTR6255 (NL6117). Registered February 8th, 2017 - retrospectively registered.

## Background

Adolescents with Autism Spectrum Disorder (ASD) are frequently referred to social skills training interventions. Limitations in social communication are the defining features of ASD, which particularly form a challenge during adolescence, when social skills become crucial for social inclusion. Several studies [1, 2] indicated that adolescents with ASD experience more negative social outcomes, such as fewer friends, little support from their classmates, very limited involvement in social activities and increased peer rejection compared to adolescents with intellectual disability or speech/language disabilities. Another study found that participants with ASD spent more time in private behaviors, and spent less time in cooperative interaction than typically developing adolescents [3].

Those with ASD who are cognitively able have increased awareness and insight in their impairments in social skills. Consequently, these impairments possibly have a negative impact in this group, resulting in greater functional impairment and poorer quality of life [4, 5]. Yet, few evidence-based, culturally specific, interventions that improve social functioning are available for cognitively able adolescents with ASD [6].

### Social skills training

Social skills groups are common interventions for those with ASD and average to above-average cognitive ability [7]. Earlier studies focused mainly on children with ASD aged 7 to 12 with average or above average cognitive ability, and were mostly carried out in the US [6]. More recently, research focused on assessing the effectiveness of treatment approaches for adolescents with ASD [8–12]. In these studies, empirical support was found for The Program for the Education and Enrichment of Relational Skills (PEERS®), which is a parent-assisted, manualized social skills training program specifically developed for cognitively able adolescents with ASD at the University of California, Los Angeles (UCLA) [13]. PEERS® might be especially effective since it is specifically aimed at maintenance of training effects through the involvement of parents as models of social instruction and real life social coaching [14]. See Table 1 for a summary of previous studies and their findings.

**Table 1:** Overview of previous studies on the effectiveness of the PEERS® parent-assisted curriculum for adolescents

Author	Design	N	Summary of significant findings per outcome measure
**Laugeson et al. 2009** [15]	RCT^a^: Waitlist control group	33	**Adolescent report:** Improved social knowledge and social contacts **Parent report:** Improvement in social skills
**Laugeson et al. 2012** [14]	RCT: Waitlist control group	28	**Adolescent report**: Improved social knowledge, social contacts **Parent report:** Improvement in social skills and reduction in social impairment
**Schohl et al. 2014** [16]	RCT: Waitlist control group	58	**Adolescent report:** Enhanced social knowledge, social contacts and declined social anxiety. **Parent report:** Reduction in social impairment as measured by the SRS
**Yoo et al. 2014** [9]	RCT: Waitlist control group	47	**Adolescent report:** Improved social knowledge. **Parent report:** decreased ASD symptoms on the Autism Developmental Observation Schedule (ADOS) and decreased depressive symptoms
**Mandelberg et al. 2014** [17]	Pre- posttest design with follow-up	53	**Adolescent report:** Improved social knowledge and social contacts (1-5 year follow-up) **Parent report**: Social functioning improvement and maintenance after (1-5 year follow-up)
**Dolan et al. 2016** [18]	RCT: Waitlist control group	58	**Adolescent report:** Enhanced social skills knowledge **Observational measure:** Improvement on behavioral observation (CASS) on subscale vocal expressiveness
**Rabin et al. 2018** [10]	RCT: Waitlist control group	41	**Adolescent report:** Increased social get-togethers, greater empathy and had more knowledge of social skills. **Parent report:** Improved social skills and reduced autism symptomology. The effects maintained at a 16-week follow-up assessment. **Observational measure:** Showed more social behavior (e.g. heightened engagement and question asking in conversations with a unknown peer)
**Schum et al. 2018** [11]	RCT: Waitlist control group	72	**Parent report**: Improved social skills knowledge, reduced autistic mannerisms and improved social functioning after intervention, replicating these results for the delayed treatment group

^a^*RCT* randomized controlled trail

All studies mentioned in Table 1 did not include an active control condition in their randomized controlled trials (RCTs). Instead, waiting list groups were used to assess the effectiveness of the intervention. Yet, these results underline the effectiveness of a protocolized social skills training, when culturally adapted to the specific cultural settings and demands. There is a large variability across cultures when it comes to social skills and social behavior [19]. Hence, to be optimally effective, the content of social skills interventions in mental healthcare must accommodate local cultural customs. Cross cultural adaptation and validation of such interventions is therefore of high importance [19]. Interventions focusing on improving social skills should be adapted to the specific needs of diverse populations, their cultural customs, and their habits. Cultural specificity of social skills includes, among others, variation in facial expressions, use of language, nonverbal and emotional cues as well as leisure time activities.

The PEERS® intervention was not yet available in the Dutch language. Responding to the need for such a programme within the Dutch mental healthcare system for adolescents with ASD, we translated and culturally adapted the PEERS® curriculum. The cultural adaption process is extensively described elsewhere [20], and was based on the recommendations of a previous study describing a cultural adaption and translation of the PEERS intervention [9]. In brief, cultural adaption and translation (including back translation) was done by 15 mental healthcare and school professionals, in consultation with the original author of the PEERS curriculum, Dr. Laugeson, The Dutch PEERS intervention was first tested during a pilot study that included 22 adolescents to assess if the adaption was successful. Given the promising results from the pilot study, we initiated the ACCEPT RCT study.

The first objective of the ACCEPT study is to investigate the effectiveness of the Dutch PEERS® intervention, by performing a randomized controlled trail, utilizing an active control condition (a psychoeducational program on several adolescent developmental issues, i.e. the Regulation, Organization and Autonomy Didactics training, ROAD). Secondly, we aim to explore potential mechanisms of effectiveness, i.e. a) mediators and b) moderators. Finally, the study uses an observational and behavioral measure of social competence, the CASS [21] similar to previous studies [10, 18], but extended with reports on the social skills of the participants by the participants and the confederates. Thus, besides investigating whether the Dutch PEERS® intervention is effective, we extend previous studies by implementing an active treatment control group and exploring which moderators and mediators influence the effectiveness of the intervention.

Below we elaborate on the methodological considerations that led to this design.

### Active treatment control condition (ATCC)

Previous studies on the parent assisted version of the PEERS® intervention used a waiting list condition as a comparison. Yet, to make sure that the experimental intervention is more effective than general effects of mental health care (i.e. receiving attention from professionals, being part of a treatment group, etc.), a preferred design is a comparison between the experimental intervention against an active control condition [22]. Therefore, extending previous studies, we compare the parent assisted version of PEERS® against an active treatment control condition.

#### Mediators

Our understanding of how the intervention works is limited. Knowledge of the specific mechanisms through which interventions work is important to further improve the effectiveness of treatments and guide the focus of interventions once they are implemented in clinical practice [23]. Mediators are variables that explain the relationship between two other variables (intervention and effect) and could themselves thus be influenced by the intervention, unlike moderators [24]. In this study, we explore potential mediators, specifically factors that are involved in social learning [25]. These factors (i.e. social knowledge, social cognition, social contacts, social anxiety and social coaching which are specified below) are often (innately) limited in individuals with ASD [26]. However, despite these initial limitations, the natural social learning process can still be stimulated. In the PEERS® intervention, the typically unconscious social learning process is now consciously being activated; spinning the so-called ‘social learning cycle’ [25].

During the PEERS® training, adolescents are first taught about social skills using concrete behavioral rules, thereby improving their *social skills knowledge*, our first possible mediator.

Subsequently, Socratic questioning is used to consciously apply this knowledge, and to improve the *social cognition* of adolescents with ASD. Social cognition entails a person’s ability to take the perspectives of others, accurately process the verbal and non-verbal signals of others, and subsequently modify one’s social behavior to the social situation. In PEERS®, perspective taking questions are repeatedly asked to stimulate the participants’ tendency to think about the other persons’ perspective in social situations.

Then, to ensure further application of the learned social knowledge and social cognitive strategies, homework assignments are another putative active ingredient of PEERS®, that promote *social contacts*.

In these actual get-togethers with peers, participants apply, practice, and fine-tune their social skills. Moreover, at a less conscious level, these social contacts can be considered exposure, which in turn can reduce *social anxiety*. Research shows that cognitively able adolescents with ASD often experience high levels of social anxiety [27], which can reduce the tendency to get into contact with peers. Thus, social anxiety should be reduced in order for advances in social contacts and skills to take place. Symptoms of social anxiety decreased in adolescents who received the PEERS® training, but whether this reduction in social anxiety is also related to improvements in social skills remains to be investigated [16].

Finally, to promote and facilitate these crucial social contacts, parents play an important role as a social coach. By assisting and motivating their child to apply the social skills during social contacts in daily life, and by providing performance feedback, parents can play a vital role in making sure that social contacts and fine-tuning of social skills are remained. However, parents should also feel competent as a social coach to be able to properly perform this vital role. Therefore, *parental feelings of competency* may also indirectly affect the outcomes of the adolescents, and are therefore considered as a mediating factor.

#### Moderators

In addition to investigating the effectiveness of the intervention, it is important to know for whom the intervention works best and under which circumstances. Such variables are moderators, i.e. variables that influences the strength of a relationship between two other variables (intervention and effect). Moderators entail patient characteristics, and are therefore preexisting to any intervention [23]. To date, we know little about the moderators of the PEERS® training. Identifying possible moderators could, in the future, help clinicians to predict which patients will be most responsive to the intervention. In addition, sub-groups could be identified from responder versus non-responder analyses, providing valuable prognostic information for future treatment outcome.

Previous studies have suggested some possible moderators for social skills treatments in individuals with ASD in general, such as *sex, age, cognitive ability*, *ASD severity, prior treatment and medication use*, but research is scarce [28]. Therefore, in the current study, we will study the role of these potential moderators.

### Observational outcome measure

Progress in developing effective treatment approaches has been delayed by a lack of sensitive and valid measures to assess meaningful improvements in social functioning [29]. Previous studies on the effectiveness of PEERS® have relied predominantly on parent and/or teacher ratings of social skills as the primary outcome [9, 14–17]. However, a logical consequence of parent involvement in the intervention is that parent-reports might be biased positively or negatively through their expectations and investment of energy and time. As a sign of this potential bias, studies that collected ratings of social skills from teachers indeed showed fewer effects, but these studies were limited by small sample sizes (largest *n* = 41 [14];). To overcome this methodological obstacle, recent research [10, 18], used a behavioral observation measure to more objectively assess social skills. These studies utilized the Contextual Assessment of Social Skills (CASS), an observational measure of social conversational skills, specifically developed for adolescents and young adults with ASD. This measure makes use of an observation of the adolescent’s social behavior, scored by a rater who is blinded for condition and time-point, and was validated as an outcome measure of social skills [21]. Intervention related improvements were found [10, 18] in this observational measure of social skills, providing evidence that the CASS probably is a useful tool to determine whether social skills targeted in treatment generalize beyond the treatment context.

In conclusion, although the evidence-base for PEERS is growing, the efficacy of PEERS® has not yet been established in a trial using an active treatment control group. In addition, little is known about possible moderators and mediators. Therefore, this manuscript describes an RCT that examines the treatment efficacy of the Dutch version of PEERS® utilizing an objective primary outcome measure of social skills.

## Methods/design

### Participants

Adolescents with ASD and their parents are included and assessed between January 2017 and October 2019. Inclusion criteria are: ASD diagnosis (DSM IV or DSM V), aged 12–18 years old, a total and verbal IQ > 70 (assessed with WISC-lV or WASI), motivated to participate (adolescent as well as his/her parents, assessment trough interview), and currently enrolled in secondary education. Participants were excluded if they met one of the following criteria; A history of major mental illness (e.g., schizophrenia, bipolar disorder, or other types of psychotic disorders), and any visual, hearing or physical impairments that prohibited the participation in the intervention.

Retention of participants is done by weekly contact between participants and trainers/study staff. At follow-up, participants receive a €30 gift voucher for their participation in the assessments.

### Sample size calculation

The sample size for this study was determined by power calculations using G*Power 3.1 [30] based on the mean and standard deviation of post-treatment results of the primary outcome measure, the CASS. For this, we used the information from a previous study [18]. To detect a difference of a moderate effect size (*d =* .50) between the experimental condition and the control condition with a power of .80 and an alpha of .05, we need at least 64 participants in each condition (total *n* = 128). To take into account a non-response/drop-out rate of ~ 15% (estimation based on a previous RCT in this population in this center [31];), we aim for a total of 150 participants.

### Intervention conditions

#### Experimental condition: PEERS® parent-assisted curriculum for adolescents

The manualised parent-assisted social skills intervention PEERS® [13] was developed to target an improvement in social skills in cognitively able adolescents with ASD. In 14 weekly session (90 min), adolescents are trained in specific social conversational and friendship skills. The intervention addresses crucial areas of social functioning for adolescents, including reciprocal conversational skills, choosing appropriate friends, the appropriate use of humor, peer entry skills, hosting get-togethers, as well as handling rejection, disagreements, and rumors or gossip [13]. Under the guidance of one or two trained (formal 3 day training) mental healthcare professional(−s), adolescents learn skills through concrete rules and role-play. Application, rehearsal and generalisation of the learned skills is enforced by homework assignments, review of the execution of these assignments during the sessions and parent-involvement. Parallel parent sessions take place in a separate room, and focus on supporting and coaching their child in executing their newly learned social skills and applying them in homework assignments and daily life. Instructors of the parent sessions were also fully certified PEERS providers. At the end of each session, parents and adolescents reunite, a review of the session is held to summarise and consolidate the key information and homework assignments are scheduled mutually to warrant completion.

#### Treatment control condition: ROAD

As an active treatment control condition, the ROAD (Regulation, Organisation, Autonomy Didactics) intervention is used. This intervention was based on the *care as usual* for adolescents with ASD at Yulius, the mental healthcare facility where the RCT was initiated. ROAD was implemented in this RCT to control for non-specific treatment effects. The intervention is manualized, fidelity checks are implemented and clinicians received specific training in order to provide the intervention.

ROAD mainly provides psychoeducation on a wide range of adolescence-related themes. In ROAD, the evidence-based training programme Tackling Teenage [17] (which is adapted to a group format and is already in use for several years) is supplemented with content from PowerCoaching (publication in preparation) to construct a similarly sized protocol format as the PEERS® intervention. The ROAD intervention provides psycho-education on developmental challenges that arise during adolescence (e.g. identity/self-acceptance, autonomy in planning activities/school-work, physical appearance and changes, regulating emotions, developing friendships, and solo/partnered sexual activities and boundaries). It is built up from more general themes (relatively easy to discuss in a group of peers) to more intimate themes (better discussion possible once group-safety is established). The main approach in the ROAD intervention is providing didactics/psycho-education, i.e. transferring knowledge and experiences in a training setting to promote application in real life through home-work assignments. Parents receive the information and assignments by e-mail, to promote help with completion of the assignments. The assignments focus on applying the knowledge in daily life, yet actual training of particular skills in session is not part of the training (no role plays). Didactic material is provided through a workbook for patients. Trainers explain the didactic material, and start a group discussion to involve participants to actively process the information. Since one session focusses on making friends and includes a homework assignment that promotes to start a conversation with an unfamiliar peer, social conversational skills are promoted as part of this intervention. Yet, this treatment target in included in a much wider range of targeted knowledge/skills, and is not as intensively trained as in PEERS.

Groups in both interventions consist of 4–10 adolescents, under the supervision of at least 1 certified and experienced clinician and accompanied by another clinician or coach (e.g. master level psychology student, who had a minimum of 6 h formal training in the intervention). PEERS trainers were not instructed on the ROAD intervention and vice-versa, to avoid cross-over of interventions. Instructors of both PEERS and ROAD received intervention specific formal training. Each session lasts 90 min. The outlines of the PEERS® and ROAD sessions are constructed in a similar way, i.e., homework review, didactic lesson, practice (PEERS®) or discussing didactic lesson (ROAD), and homework assignments for next week. In the PEERS® intervention, parents are involved in 14 parallel social coaching sessions. They are trained in the PEERS® curriculum to learn to support their child. In the ROAD intervention, parent involvement is less intensive. Parents only receive, via email, an outline of the didactics, homework assignments and a summary after each of the sessions to be up to date about what their son/daughter had discussed in the session.

### Study design

The current study is a two-arm parallel group randomized controlled trial (RCT) where each participant is randomly assigned to either the experimental condition (the PEERS® intervention) or the active treatment control condition (ROAD). The study is carried out within 3 mental healthcare institutions that provide specialised in- and outpatient care for individuals with ASD, i.e. Yulius Mental Healthcare, De Jutters Child and Adolescent Psychiatry (South-western provinces of the Netherlands), and Jonx Mental Healthcare Groningen (North-eastern provinces of the Netherlands), ensuring geographical diversity in our sample. In these centres, adolescents are referred to the ACCEPT study when an out-patient mental healthcare specialist (i.e. psychologist/psychiatrist/pedagogue) indicates that both PEERS® and ROAD are appropriate intervention for the adolescent, and when both the adolescent and his/her parent(s) are motivated to take part in all 14 sessions. In addition, the Erasmus University Medical Centre Rotterdam, Lucertis and BOBA (also mental healthcare facilities with specialized autism in- and outpatient care) act as referral sites for the study, i.e. clients from these centres are referred to Yulius or the Jutters if PEERS®/ROAD is indicated. Moreover, adolescents and their parents can apply for participation in the study themselves after reading the information on websites, leaflets/posters or on social media, via referral by their general practitioner. After referral, adolescents and their parents are contacted by phone to inform them about the study and to get permission to send them the more detailed study information. After a week, potential participants are contacted for a second time to see if they have additional questions after reading the detailed information, and to check in- and exclusion criteria. Subsequently, the adolescent and his/her parent(s) are invited for an intake appointment (60 min). The goal of the intake is to assess their motivation (through a semi-structured interview), to check whether their treatment goals is in line with the interventions, and to further inform them about the procedures and check their understanding of the information that has been provided. Written informed consent is obtained from all adolescents and their parents by trained study personnel. This study is approved and guided by the medical ethical commission of the Erasmus Medical Center, Rotterdam (MEC-2016-357, protocol version 6.0, February 8th, 2018). All researchers have participated in training sessions addressing good clinical practices. All essential documentation and trial records are stored by Erasmus MC and participating local sites in conformance with the applicable regulatory requirements with access to stored information restricted to authorised personnel. Monitoring of the study progress was done yearly. Further information is available upon request.

**Fig. 1:**
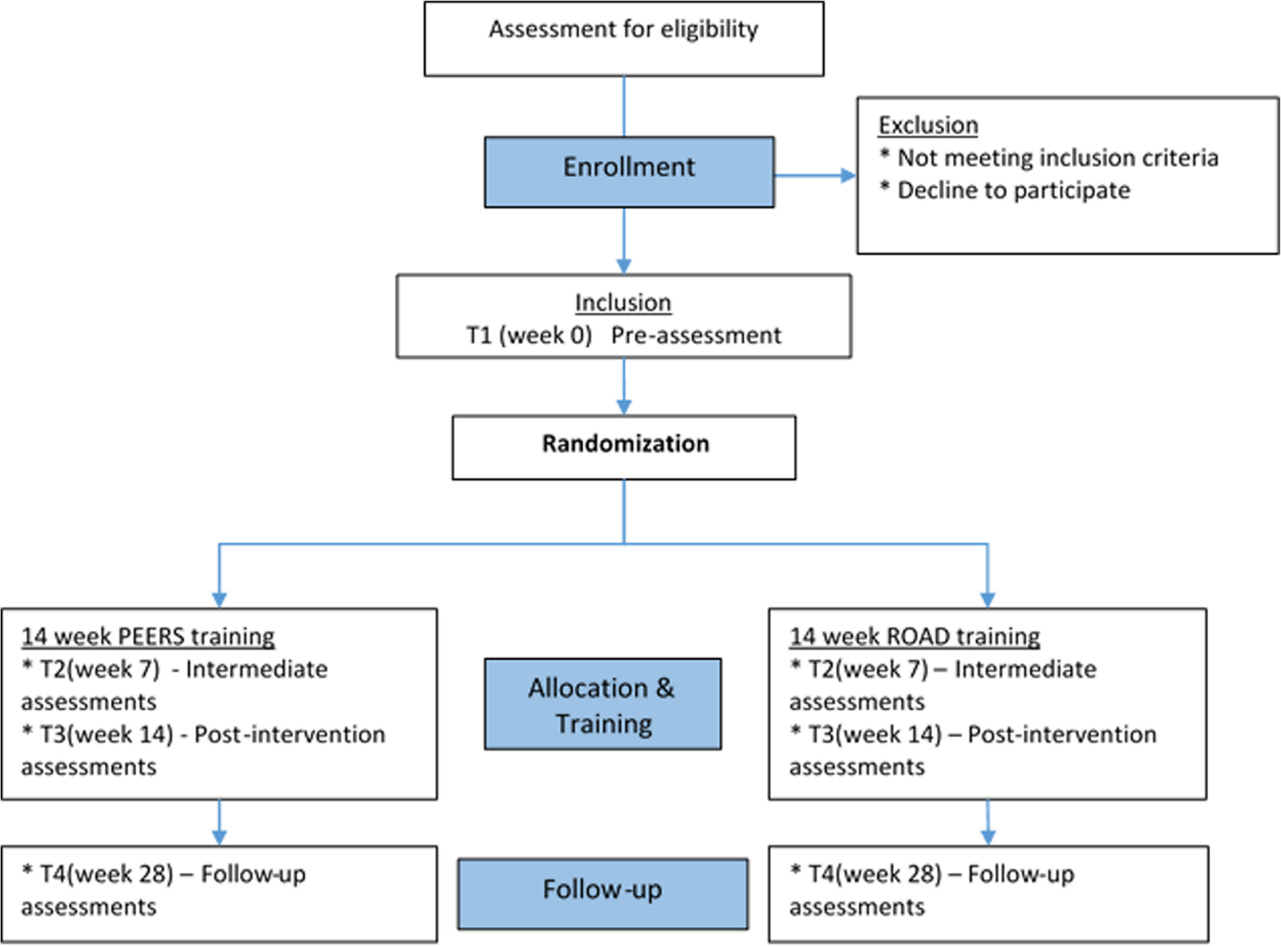
ACCEPT Flow Diagram

Four assessments are conducted, i.e. prior to the randomization (baseline: T1), halfway during the intervention (week 7: T2), directly at the end of the 14-week intervention (week 14: T3), and a 14-week follow-up after the intervention (week 28: T4). A schematic diagram of the trial design, procedures, and stages of data collection is provided in Fig. 1 and Table 2.

**Table 2:** Overview of assessments and instruments

Instrument	Domain	Format	Subject	Duration	T1	T2	T3	T4
**Adolescent**								
CASS	Social skills	Observation (video recorded)	Adolescent	10	v	–	v	v
SRS-2	Social skills	Questionnaire	Adolescent	15	v	v	v	v
SSIS-RS	Social skills	Questionnaire	Adolescent	15	v	v	v	v
TASSK-R	Social knowledge	Questionnaire	Adolescent	10	v	v	v	v
QSQ-A	Social contacts	Questionnaire	Adolescent	5	v	v	v	v
BFNES	Social anxiety	Questionnaire	Adolescent	5	v	v	v	v
TUSC	Social cognition	10 Videos	Adolescent	20	v	–	v	v
Satisfaction	Treatment satisfaction	Questionnaire	Treatment	5	–	–	v	–
**Parent**								
Registration form	Work and educational status, ethnicity, medication use and prior treatment	Questionnaire	Adolescent	5	v	–	–	–
SSIS-RS	Social skills	Questionnaire	Adolescent	15	v	v	v	v
SRS-2	Social skills	Questionnaire	Parent	15	v	–	–	–
QSQ-P	Social contacts	Questionnaire	Adolescent	5	v	v	v	v
PSOC	Parenting feelings of competency	Questionnaire	Parent	5	v	v	v	v
Satisfaction	Treatment satisfaction	Questionnaire	Treatment	5	–	–	v	–
**Teacher**								
SSIS-RS	Social skills	Questionnaire	Adolescent	15	v	v	v	v
SRS-2	Social skills	Questionnaire	Adolescent	15	v	v	v	v

*CASS* Contextual Assessment of Social Skills, *SSIS-RS* Social Skills Improvement System-Rating Scale , *SRS-2* Social Responsiveness Scale, *TASSK-R* Test of Adolescent Social Skills Knowledge, *QSQ-A or P* Quality of Socialisation Questionnaire- Adolescent or Parent report, *BFNE* Brief Fear of Negative Evaluation, *TUSC* Test of Understanding Social Conventions, *PSOC* Parenting Sense of Competence scale

### Procedures

Adolescents and parents complete all tasks and questionnaires on paper except for the intermediate assessment (T2) which are completed online. Teachers complete questionnaires online at all time-points. Table 2 provides an overview of all instruments and time-points.

Our main primary outcome is the Contextual Assessment of Social Skills (CASS) and is administered to measure social skills. Secondary endpoints of primary outcome domain social skills are the Social Responsiveness scale (SRS-2) and Social Skills Improvement System (SSiS-RS). In addition, various instruments are used to detect possible mediators, which include: enhanced social knowledge (storage of facts in declarative memory) as measured by Test of Adolescent Social Skills Knowledge (TASSK). This is done because learning stages before improvement in social skills include enhanced social knowledge. Furthermore, improvement in insight of social situations (applying this knowledge when interpreting situations with other people, perspective taking skills), or social cognition, is measured by the Test for Understanding Social Conventions (TUSC), social anxiety is measured with the Brief Fear of Negative Evaluation (BFNE-II), increasing social learning experiences are measured with the Quality of Socialisation Questionnaire (QSQ) and the Parenting Sense of Competence Scale (PSOC) is administered to assess self-reported parenting competency.

During the baseline, post- and follow-up assessment, the parents and adolescents are escorted to different rooms. Assessments take place at the mental healthcare center where the adolescents receive the intervention. A test leader is present in both rooms to coordinate the assessments, give instructions and answer questionnaires if needed. The parents are asked to complete the questionnaires, which takes approximately 50 min. The adolescents first complete the social cognition task (TUSC; see 2.7). The test duration is approximately 20 min.

After the social cognition task, the adolescents are asked to complete several questionnaires, taking around 50 min. In parallel, each adolescent is asked to have a 3-min conversation with an unfamiliar typically developing peer (CASS) in a separate room (see 2.7). A camcorder is placed to record these interactions.

At post-intervention (T3) assessment, additional treatment satisfaction questionnaires are administered to parents and adolescents to gather qualitative data on their satisfaction of the intervention.

Adherence to treatment protocol is monitored by trained research assistants in both parent and teen PEERS® groups and ROAD teen groups through weekly fidelity sheets outlining the manualized intervention.

In both intervention groups, compliance with homework assignments and attendance is recorded for teens and parents. Fidelity and compliance information is collected to enable us to control for dose-response effects, and thus will be considered in the analyses as co-variates.

### Randomisation and blinding

Randomization takes place after the first assessment, so that the initial assessment will not be affected by knowledge on which program participants will receive [23]. Blocked randomization is used to ensure close balance of the numbers in each intervention group at any time during the trial. Allocation to conditions is determined by computer-generated randomization. Researchers who are involved in the analyses are blind to the treatment allocation. In addition, staff members involved in the CASS coding and analyses are kept blind to the intervention assignment and the time point of the assessment being coded. Participants are instructed not to discuss the intervention with the confederate of the CASS. Participants are also asked not to tell their teacher which intervention they receive. It is not possible to blind the participants or the clinicians providing the treatments. However, we emphasize to intervention staff and participants that each intervention adheres to enrichment of the social-emotional development and each intervention is presented by experts to provide the adolescents the tools to promote their independence as much as possible. In addition, all staff members and participants are kept blind to the results of the assessments.

### Outcome measures

#### Primary measure of primary outcome – social skills

*The Contextual Assessment of Social Skills (CASS)* [21] is an observational measure of social and conversational skills developed for cognitively able adolescents and young adults with ASD. During the CASS, participants undergo a 3-min conversation with a confederate (i.e., an unfamiliar, opposite sex, similarly-aged peer without ASD). The CASS guidelines describe that all interactions should be with a similar aged, opposite sex peer. Participants will interact with confederates of the opposite gender, as one of the social developmental tasks in adolescence is to engage in interactions with the opposite gender. Opposite gender interactions become more prominent in adolescence, since the desire of a romantic relationship develops, and previous same gender play-focussed interaction fade out [21].

At each assessment wave, a different confederate is introduced to make sure the observed conversation is based on initiating contact with an unfamiliar peer. Confederates are typically developing adolescents that are recruited through advertisements in schools and word of mouth amongst colleagues. Confederates receive reimbursement for their time (€25 per assessment wave) and a three-hour formal training precedes their participation in the RCT in which they receive instructions and practice regarding their behavior (see below).

For each participant, the test leader reads the instruction to the participant outside of the room where the confederate is sitting. The participant is asked to fill out the level of confidence they feel just before they enter the room. Subsequently, the participant enters the room. The participant is instructed to start the conversation, while the confederate is instructed to wait for the participant to start the conversation, this ensures that the participant is allowed to demonstrate initiation skills. After 3 minutes, the test leader knocks on the door as a sign to the participant to end the conversation. Again, the confederate is instructed to leave the finishing to the participant in order to allow them to demonstrate their finishing-off skills. Subsequently, the participant as well as the confederate are asked to complete a brief accompanying questionnaire about how they experienced the conversation (the Conversation Rating Scale - CRS [32]; that comes with the CASS). In this way, we obtain the self-perceived competence and confidence of the participant as well as a peer-report on the social behavior of the participant during the conversation. The CRS originally covers items about perceived interest, friendliness, conversational flow, and sense of boredom/distance. Three items about self-confidence and the alleged perspective of the conversational partner on the conversation were added to align more closely to the learning goals of the PEERS program (i.e. taking on the perspective of others). In their reports, confederates are encouraged - and explained that it is important - to give their true opinions. During the conversation, confederates are instructed to demonstrate moderately interested behavior (i.e. a level of interest of 7 on a scale from 0 to 10). They are instructed to make sure that the appropriate nonverbal cues are displayed (i.e. level of eye contact, openness of posture, and amount of gestures and smiling). Confederates are told to be supportive of the conversation, but not to carry the full conversational load. They should mainly follow the topics of conversation the participants introduce, and leave the introduction of new topics to the participants, in order to allow the participants to initiate new topics. Confederates are allowed to speak no more than 50% of the time and wait 10 s after the examiner leaves the room for the participant to initiate the conversation. Confederates can use standard prompts for initiation if necessary (i.e. How was your weekend?). If the conversation stops for a while, confederates are instructed to wait 5 s before reinitiating the conversation, to first allow the participant to restore the conversational flow. The conversation is videotaped for later coding. Codes are assigned based on a) the participant’s and b) the confederates verbal and non-verbal behaviors, across nine primary domains; Asking Questions, Topic Changes, Overall Involvement, and Overall Quality of Rapport; Social Anxiety, Kinetic Arousal, Vocal Expressiveness, Gestures, and Positive Affect, and four additional domains; Initiating the Conversation, Finishing the Conversation, Dominating the Conversation, and Long Silences. These additional domains are introduced to fit more closely to the PEERS learning objectives. Most domains are rated on a Likert scale ranging between 1 (low) and 7 (high) [21, 33], although Asking Questions, Topic Changes and Long Silences are count scores. Within the Asking Questions domain we distinguish between a) Initiating Questions and b) Follow-up Questions, more in line with the PEERS learning objectives. Rating is performed by trained study personnel (e.g. Master students) who achieved 80% consensus on training recordings. The videos are also used for scoring the behavior of confederates, since differences in social skills of the confederates were found to significantly differ between confederates [18]. In this way, we are able to control for social behavior of the confederate. A previous study showed an inter-rater reliability for CASS using intra class correlation coefficient (ICC) for the nine items separately. ICCs ranged from .50 to .97 with a mean value of .68. Internal consistency for the nine items was high (alpha = .83) [21]. The CASS was also found to be a sensitive measure and is able to detect differences in behavior in social context [18, 21].

#### Secondary measures of the primary outcome - social skills

Because the CASS is a three minute observational measure, we also collect multi-informant information about the past two weeks.

*Social Skills Improvement System-Rating Scales (SSIS-RS)* is a questionnaire for the assessment of social skills at home, in the classroom and in interactions with PEERS® [34]. The SSIS-RS is administered to parents, teacher and adolescents. Each version consists of 46 items. The study uses social skills subscales, i.e. communication, assertion, empathy, engagement and self-control. It takes 15 min to complete and has shown to be sensitive to change in social skills among high functioning adolescents with ASD in the PEERS® Program [35].

*Social Responsiveness Scale-version 2 (SRS-2)* is a 65-item questionnaire with 4-point scale from 0 (not true) to 3 (almost always true) and a total score ranging from 0 to 195. It measures the severity of social impairment [35] and is completed by parents and teachers. The SRS-2 [36] is suitable for children and adolescents aged 4–18 years and also has an acceptable model fit with the two-factor structure of ASD as defined by DSM-5, being social communication impairment and restricted, repetitive behavior [37]. It provides information for five specific symptom domains (i.e. social awareness, social cognition, social communication, social motivation, and autistic mannerisms). The Dutch version of the parent report SRS-2 demonstrated high internal consistency (Cronbach’s alpha ranged from .92 to .95, good convergent validity (r = .63 with the ADI-R) and was able to differentiate between children with ASD and from the general population [38]. Parent’s autistic symptoms is measured using the SRS Adult at baseline.

#### Potential mediators influencing social skills

##### Social knowledge

*Test of Adolescent Social Skills Knowledge (TASSK)* consists of 30 items which assess changes in participants knowledge of the specific social skills taught during the PEERS® program. The adolescent answers questions related to the didactic lessons by choosing the best options from two possible choices. Scores range between 0 to 26 with higher score reflecting greater knowledge of adolescent social skills. The test is sensitive to treatment effects and has a Cronbach’s alpha of 0.56 [13].

##### Social cognition

*The Test of Understanding Social Convention (TUSC)* is a measure specifically developed for the purpose of the present study to assess particular aspects of social cognition (i.e. perspective taking, social prediction) that are more closely related to what adolescents learn during PEERS® training (i.e., a proximal outcome). The TUSC consists of ten video-clips, similar to those used in PEERS® to demonstrate the social rules/conventions. In each video clip adolescents interact in naturalistic everyday situations and a social rule or convention that is taught in the PEERS® training is central, for example ‘Don’t get too personal at first’ or ‘Don’t stand too close’, with the characters either violating or following this rule/convention. Each video-clip is followed by three multiple-choice questions: a) how was this interaction for person A? b) what do you think person A thought of person B?, c) do you think person A will want to talk to person B again?, to assess the ability of the adolescent to take on the perspective of others and predict their future behaviors. These questions are similar to those used during the intervention as part of Socratic techniques to enhance mentalization.

##### Social contacts

*Quality of Socialization Questionnaire – Adolescent/Parent (QSQ-A / QSQ-P)* is a 12-item self-report and parent-report measure to assess the frequency of adolescents get-together with PEERS®, number of friends involved and the level of conflict during get-togethers over the previous month [13]. Previous studies have shown that the ASD group that received PEERS® showed an increase in social contacts over time compared to a waiting list control group [9, 14–17].

##### Social anxiety

The *Brief Fear of Negative Evaluation scale-II (BFNE-II* [39]*;* is a revised version of the Brief Fear of Negative Evaluation scale *(BFNE* [40]*;)*. It is a self-report questionnaire consisting of 12 items reflecting fear of negative evaluations, which is central to social anxiety. The questionnaire correlates high with other measures of social anxiety and is sensitive to treatment-based changes. Participants indicate how much each item applies to them on a Likert Scale ranging from 0 (“Not at all characteristic of me”) to 4 (“Extremely characteristic of me”). In past research, the BFNE-II has demonstrated good psychometric properties with an alpha coefficient of 0.95 [41].

##### Parental feelings of competency

The *Parenting Sense of Competence Scale (PSOC)* is a parent-report questionnaire, consisting of 16 items that measures parenting self-efficacy, parental feeling of competency, parental capacity of problem solving, and familiarity with parenting [42]. The questionnaire includes a six-point Likert scale ranging from 1 (strongly disagree) to 6 (strongly agree). Examples are “I meet my own personal expectations for expertise in caring for my child” and “My talents and interests are in other areas, not in being a parent”. The PSOC has good divergent construct validity for the parenting satisfaction subscale of the PSOC. Internal consistency is good [43, 44].

#### Potential moderators

Participant characteristics that are considered putative moderators of treatment effect, assessed at baseline, are: ASD severity, cognitive ability (intelligence: IQ), sex, age, concurrent medication/ treatment and previous social skills treatment [28] and presence of autistic symptoms in parents. Measures of ASD severity and IQ are described in more detail below. To assess the remaining moderators (i.e. sex, age, concurrent medication/treatment and prior social skills treatment), caregivers are asked to complete a questionnaire assessing these characteristics.

In order to further formulate useful clinical recommendations about effectiveness of the intervention on possible subgroups, we plan to carry out a responder analysis on the primary outcome measure, CASS. By identifying responders vs. non-responders, more information is gained about for whom the intervention is best suitable.

##### ASD symptom severity

To assess ASD severity, the Autism Diagnostic Observation Schedule Second Version (ADOS-2 [45]) is used. The ADOS is a semi-structured observational assessment which aims to elicit communicative and social behaviors. The observation results in a total calibrated severity score (controlling for language level and age), comprised of two sub-scales: Social Affect (SA) and Restrictive, Repetitive behavior (RRB).

The ADOS-2 has excellent test-retest reliability (0.82) and inter-rater reliability (0.92). If the ADOS-2 has been administered in the past 5 years, these scores are distracted from the patients file with permission from the parents. If the ADOS is not available, a trained and licensed clinician will administer the ADOS.

##### Cognitive ability (IQ)

If available, information on IQ is extracted from the patients file with permission from the parents. If IQ has not been assessed within the last five years, the Wechsler Abbreviated Scale of Intelligence, suitable for individuals from 6 to 90 years (WASI [46]) is administered to assess the cognitive ability (IQ) of the participant. The WASI is a clinically useful screening instrument to assess intelligence with good convergent and discriminant validity compared to Kaufman Brief Intelligence Test [47].

### Data analysis

The primary outcome of this study is social skills improvements as assessed via the CASS. Hierarchical linear modeling (HLM [48]) will be performed to analyze the effects of time (level 1) and condition (level 2) on the outcome on the CASS. Time (indexed pre-treatment (week 0), during treatment (week 7), post-treatment (week 14) and the follow-up (28 weeks)) will be entered as the level-1 predictor. Treatment condition (PEERS® or ROAD) will be entered as a level-2 variable to explain the variation in growth trajectories [48]. Specifically, we are interested whether the predicted increase of scores over time in the PEERS® treatment condition will be greater than in the control condition, by investigating a time*condition interaction. To avoid drop-out effects, an intention-to-treat analysis will also be performed.

First, we will perform preliminary analyses (i.e. correlations) to check whether ‘third variables’ such as demographic/diagnostic, group dynamic variables and/or fidelity/compliance variables, are related to the main outcome measures. If so, we will control for these variables in the main analysis by adding them as covariates. Fidelity data will be used primarily as descriptive data to assess the ‘dosage’ participants were exposed to (% of protocol that was covered). Confederate social skills (i.e. CASS scores of the confederates) are also explored as a potential covariate that might be related to the scores on the CASS of the participants, as social behaviors of the confederates could influence the social performance of the participants. Finally, group belongingness (belonging to a certain group of participants during the intervention, with i.e. (un) secure ambiance) will be explored as potential influencer on individual outcomes.

#### Mediator and moderator analysis

A variable is considered to be a mediator of the treatment effect, if changes in this variable during treatment (T1-T3) precede changes in the outcome variable. To investigate this, we will conduct latent growth curve models (LGMs) as performed by an earlier study which also used this technique for the assessments of mediators, moderators and predictors of treatment effects [49].

In all analysis (other than the primary outcome), we will control for multiple testing with the False Discovery Rate (FDR).

## Discussion

The need for evidence-based, culturally adapted social skills interventions has been highlighted by recent studies [19]. In that light, we set out to conduct a unique, large scale RCT with an active control condition to assess the effectiveness of the Dutch parent-assisted PEERS® intervention amongst cognitively able adolescents with ASD. Combined with the assessment of social skills with an observational measure, the wide geographical range of participants within The Netherlands and a wide range of secondary outcome measures for the exploration of possible moderators and mediators of the effectiveness of the intervention, make our design methodologically sound and it should allow for generalisation of results to the wider population.

However, several limitations do apply. First, randomisation of participants into two different conditions (i.e. intervention programs) could be an obstacle for referral for some clinicians or for participation for some of the participants/parents. The clinician, adolescent, and/or parents of the patient may have a preference for one of the interventions. By informing the participants and parents about the similarities and differences of both interventions and how these meet the participants needs, we hope to minimize the uncertainty. Second, randomisation outcome is communicated right after the baseline assessment, so a full week before the start of the training. However, the unpredictability of which training the adolescent receives may cause stress for some of the potential participants and/or their parents/clinicians. Third, although our predictions of recruitment rate are based on a similar study within the same institutions [30], we do realise that including the proposed sample of *n* = 150 is challenging. The study is conducted within specialized mental healthcare settings. Therefore, the possibility exists of a higher rate of refusal to participate or an increased attrition rate after enrolment in the study, because patients with more severe problems (i.e. ASD core symptoms, comorbidity and/or societal isolation) are nowadays referred to these centres. Multiple research sites and extensive intake interviews should, in part, make it possible to acquire the intended sample size. Additionally, in this study, three measures are used that were developed especially for the assessment of participants in the PEERS intervention, namely the TASSK, QSQ and TUSC. We therefore need to be careful in the interpretation of these secondary outcome measures and compare them with the more general outcome measures like SRS-2 and the SSiS-RS.

Since there were no previous studies that used an active control condition, input for effect size estimates is not available**.** Therefore, we based our effect size estimation on earlier studies that used waiting list control conditions. Yet, potentially more conservative estimates could have been used. Caution about effect sizes will be kept in mind during data analysis.

Both interventions are manualised and have a 14-week format, but differ in content and parental involvement. The active control condition has modest parental involvement (i.e. online only, not face to face) and focusses on puberty psychoeducation, thus mainly enhancing the *knowledge* of social skills of adolescents with ASD. We therefore expect to find progression in several secondary outcome measures, but marginal enhancement in social skills. By contrast, the PEERS® intervention focusses on enhancing social skills, with practical exercises and active parental involvement, targeting towards actual *behavioural change*. We therefore expect to find more favourable outcomes on enhanced social skills compared to the control condition.

Both interventions find their overlap in educating adolescents about friendships and dealing with arguments in a group setting under the supervision of mental healthcare professionals. Both interventions also contain similar strategies to enhance effectiveness as a group training: They include multiple trainers (enhanced level of attention to all group members), a fun training environment, errorless learning, structured and predictable lessons and homework assignments [50]. As such, non-specific treatment effects can be expected and should be considered when examining the results with regard to the secondary outcome measures in both the experimental and active control condition.

With this study, we expect to make a valuable contribution to the knowledge about cultural adaptions of evidence-based social skills training programmes for adolescents with ASD. We hope to add to the international evidence base regarding the PEERS® intervention, specifically shedding more light on the working mechanisms of social skills trainings by identifying mediators, and uncovering moderators related to treatment response.

## Data Availability

The datasets generated and/or analyzed during the current study are not publicly available due to ongoing data collection, but are available from the corresponding author on reasonable request after the study ends and results are published. Only authors have access to the data.

## Abbreviations

ADOS: Autism Developmental and Observation Schedule
ASD: Autism Spectrum Disorder
ATCC: Active Treatment Control Condition
BFNE: Brief Fear of Negative Evaluation
CASS: Contextual Assessment of Social Skills
PEERS®: Program for the Education and Enrichment of Relational Skills
PSOC: Parenting Sense of Competence scale
RCT: Randomized Controlled Trail
ROAD: Regulation, Organization and Autonomy Didactics
SRS − 2: Social Responsiveness Scale – version 2
SSIS-RS: Social Skills Improvement System-Rating Scale
TASSK-R: Test of Adolescent Social Skills Knowledge
TUSC: Test of Understanding Social Conventions
UCLA: University of California, Los Angeles
QSQ-A or P: Quality of Socialisation Questionnaire- Adolescent or Parent report
WASI: Wechsler Abbreviated Scale of Intelligence
